# Atlantic Salmon Alevins Experimentally Exposed to Salmon Gill Poxvirus Become Infected, With the Virus Targeting Epithelial Cells in the Gills, Oral Cavity and Skin

**DOI:** 10.1111/jfd.14127

**Published:** 2025-04-05

**Authors:** Marit Måsøy Amundsen, Haitham Tartor, Kristrun Kristþórsdóttir, Snorre Gulla, Brit Tørud, Simon Weli, Mona Cecilie Gjessing

**Affiliations:** ^1^ Norwegian Veterinary Institute Norway; ^2^ Vetaq Iceland

**Keywords:** cell tropism, challenge experiments, emerging diseases, SGPV tropism

## Abstract

Infection with salmon gill poxvirus (SGPV) can cause severe gill pathology, leading to respiratory distress and high mortality rates in salmon hatcheries, known as salmon gill poxvirus disease. While the infection has been documented in salmon at sea, broodfish and wild salmon, its occurrence in salmon alevins remains unreported. This study presents four trials aiming to infect salmon alevins with SGPV, focusing on variations in storage conditions, processing of the infectious material and cohabitation. Utilising RNAscope in situ hybridisation and PCR techniques, we show that epithelial cells in the gills, oral cavity and skin of Atlantic salmon alevins can indeed be infected with SGPV. Moreover, our findings revealed that storing the challenge material at − 20°C compromises the virus infectivity. In contrast, preservation at − 80°C retains infectivity, even in the supernatant of homogenised infected gills.

## 1 Introduction

Poxviruses are large, complex enveloped DNA viruses with a double‐stranded genome infecting a wide range of animals. But for teleost, poxvirus infection has so far only been recognised in commercially important species like Atlantic salmon (Gjessing et al., 2015, 2017), carp (Ono et al. 1986) and recently also Atlantic cod (Gjessing, Tengs, Nilsen, Mohammad & Weli, 2025). The salmon gill poxvirus (SGPV) belongs to the sub‐family of Chordopoxvirus (ChPV), with a size of 360 × 270 × 250 nm and a genome of more than 240 kb (Gjessing et al., 2015). The virus infects gill epithelial cells of Atlantic salmon ( *Salmo salar* L.) (hereafter salmon) and can cause severe gill pathology leading to salmon gill poxvirus disease (SGPVD). Transmission electron microscopy of gill epithelial cells from salmon suffering from SGPVD first identified salmon gill poxvirus in 2008 (Nylund et al. 2008). Then, the complete virus genome was sequenced in 2015 (Gjessing et al., 2015) enabling the development of diagnostic tools, including qPCR assays, antibodies for immunohistochemistry (Gjessing et al., 2015) and RNA scope in situ hybridisation (Thoen et al. 2020).

The use of these methods has shown that SGPV is widespread among Atlantic salmon in the north‐eastern Atlantic region. The virus has been identified not only in several freshwater hatcheries, but also in sea farms, broodfish farms and wild salmon populations (Gjessing et al., 2017; Garseth et al. 2018; Gulla et al. 2020). In hatcheries, SGPVD outbreaks may lead to high, acute mortalities with juvenile salmon in respiratory distress caused by extensive apoptosis of gill epithelial cells (Gjessing et al. 2015, 2017, 2018) and sometimes severe erythrophagocytosis (Gjessing et al. 2015, 2020). These severe outbreaks are typically preceded by a stressful event and have been confirmed experimentally (Thoen et al. 2020). Sub‐clinical SGPV infections with low viral loads are common and infection with SGPV in salmon at sea is often seen in combination with other pathogens (Gjessing et al., 2021) sometimes playing a part in complex gill disease (Gjessing et al., 2021). However, due to the occasional very severe outbreaks in salmon hatcheries, SGPV is considered a significant and feared pathogen in the salmon farming industry. Neither gill diseases in general nor SGPVD are notifiable diseases, making it challenging to accurately track the annual number of cases and the extent of virus‐related mortalities in salmon hatcheries. However, extensive screening has confirmed the presence of SGPV in Norway, Scotland and the Faroe Islands (Gjessing et al., 2018; Gulla et al. 2020), and in this study, also in Iceland.

Due to the lack of SGPV culture systems, experimental infection challenges are required, utilising SGPV‐infected tissue as the challenge material. In a previous study (Thoen et al. 2020), whole salmon suffering from SGPVD were euthanised and shipped overnight on ice to the research facility, serving as challenge material. The findings from that study confirmed that stress, mimicked by cortisol, in combination with SGPV infection, triggers the manifestation of severe SGPVD. However, the storage conditions of SGPV‐infected tissue for experimental infection may affect viral infectivity (unpublished data). As an accurate and reproducible infection model is essential in the research of infectious diseases, this study investigates the effects of storage temperature and tissue homogenisation on infection outcomes. Additionally, since SGPV infection in salmon alevins has not been documented, our goal is to determine whether this developmental stage can be infected and evaluate their potential as experimental models for SGPV research. Demonstrating the susceptibility of alevins to SGPV could enable their use as a continuous source of viral shedding for successful in vitro cell infection trials. Furthermore, our research on the immune response of salmon pre‐smolts to SGPV indicates that fish surviving experimental SGPV infection may exhibit reduced susceptibility to subsequent viral exposures (unpublished data). Establishing an SGPV infection model in alevins would also facilitate studies on viral infection kinetics and mortality rates in virus‐primed alevins during later developmental stages.

This study presents the first report of SGPV infection in Atlantic salmon alevins, demonstrating apoptotic, SGPV‐infected epithelial cells in gills, skin and oral cavity. We highlight the critical importance of storing the challenge material for SGPV experiments at − 80°C to ensure successful outcomes in challenge trials. Additionally, we emphasise the benefits of using homogenised challenge material for improved experimental reliability.

## 2 Material and Methods

### 2.1 Experimental Animals and Sampling Procedure

Atlantic salmon alevins (560–630 degree‐days) were obtained from the Norwegian University of Life Sciences. Since the European Food Safety Authority and the Norwegian Food Safety Authority do not require approval for experiments involving alevins (The Norwegian Food Safety Authority 2024; The Norwegian Ministry of Agriculture and Food 2015), no Forsøksdyrforvatningen tilsyns‐ og søknadssystem application was submitted for the experiments conducted in this study. The alevins were euthanised by an overdose of benzocaine (≈400 mg/L) (Benzoak vet, EuroPharma, Norway) before sampling and preserved in 10% buffered formalin (Chemi‐Teknik AS, Oslo, Norway) for histopathology and in situ hybridisation. Alevins for qPCR and RT‐qPCR analysis were preserved in microcentrifuge tubes containing 1 mL RNAlater (Thermo Fisher Scientific, Cat. No. AM7021). Due to the small size, the entire alevin was preserved, and the heads were later cut off and analysed.

### 2.2 Challenge Material

In this work, salmon suffering from SGPVD from three different facilities (sites 1–3) were used as challenge materials (CM1—CM3). Challenge materials 1 (CM1) and 2 (CM2) were sampled from two Norwegian salmon facilities, with weight measurements of approximately 160 g per fish and 180 g per head respectively. CM3 was obtained from an Icelandic facility, with approximate weight measurements of 6 g per fish. The diseased fish were euthanised and shipped on ice overnight to the research facility and were stored as described next. The challenge material was also evaluated to ensure it was free from other gill pathogens as part of the diagnostic routines at the Norwegian Veterinary Institute (NVI).

### 2.3 Challenge Experiments

**FIGURE 1:**
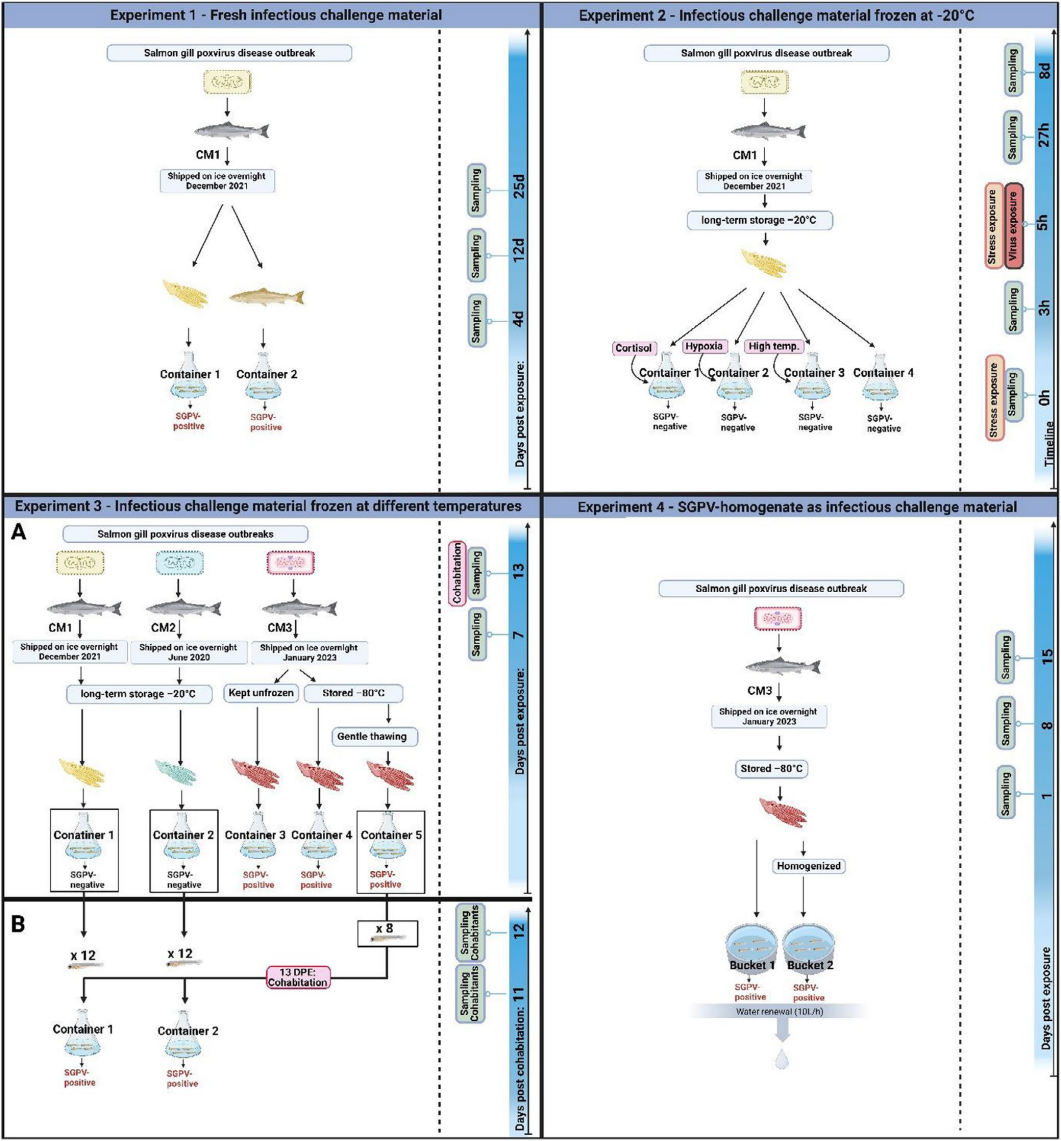
Illustration of the four salmon gill poxvirus (SGPV) challenge experiments on alevins conducted in this study. The experiments are shown in chronological order from Experiment 1 to Experiment 4, and the timeline for sampling is shown in blue vertical lines. CM1–3 are challenge materials from sites 1–3, indicated as yellow, turquoise and red gills respectively. The gills used as challenge material was unprocessed in Experiments 1–3, while CM3 was homogenised in Experiment 4. ‘SGPV‐positive’ or ‘SGPV‐negative’ indicates if the alevins were infected or not. Negative control tanks are not shown in the figure (created with BioRender.com). https://BioRender.com/x63q968.

From December 2021 to June 2023, four SGPV challenge experiments were carried out to infect Atlantic salmon alevins with SGPV. An overview of the experimental designs is shown in Figure 1.

### 2.4 Experiment 1: Alevins Exposed to Fresh SGPV‐Infected Material

In the SGPV challenge experiment published in 2020 (Thoen et al. 2020), whole salmon obtained from an SGPVD outbreak was shipped on ice overnight and used as challenge material upon arrival, that is, without freezing. To assess whether SGPV‐infected gills also can serve as challenge material, alevins were exposed to both whole infected salmon and gills dissected from SGPV‐infected salmon. A total of 60 alevins (630 degree‐days) were transferred to two 2‐L containers at 4.8°C with 30 alevins in each. One whole SGPV‐infected fish from CM1 (SGPV Ct; 21.8) of about 160 g was transferred to container 1, while alevins in container 2 were exposed to infected gills dissected from one salmon from CM1 (SGPV Ct; 20.2). After 24 h, the challenge material was removed, and the water in both containers was changed. Alevins were sampled at 4, 12 and 25 days post‐exposure (DPE) for qPCR, histopathology and in situ hybridisation work.

### 2.5 Experiment 2: Alevins Exposed to SGPV‐Infected Material Stored at − 20°C

Storage of the SGPV CM at − 20°C was suspected to have a negative impact on the infectivity of SGPV in smolt (data not shown). To investigate this further, we ran another trial using CM1 kept at − 20°C and to rule out the absence of stress as a potential explanation for negative results (stress was shown to be a key factor in experimental induction of SGPVD; Thoen et al. 2020), alevins in Experiment 2 were exposed to three different stress inducers. A total number of 480 Atlantic salmon alevins (600 degree‐days) were divided into six 2‐L containers with 80 alevins in each. The alevins were acclimatised for 3 days at 6.5°C, with aerators maintaining average O_2_ levels at 10 mg/L. The whole gills CM1 was stored at − 20°C for 3 months (SGPV Ct value after storage was 23.0) and was used in this experiment. Prior to exposure to CM1, 100 ng/L hydrocortisone (Solu‐Cortef, Cat. No. 52240) was added to container 1. In container 2, the O_2_ levels were decreased from 10 mg/L to 5.3 mg/L by removing airflow and 2/3 of the water, while in container 3, the water temperature was increased from 6.5°C to 20°C. The O_2_ level and temperature of the containers in this experiment are shown in Figure SS1. The conditions were maintained for 5 h before the stress inducers were removed (i.e., changing water in container 1, increasing O_2_ levels in container 2 and decreasing water temperature to 6.5°C in container 3), and the alevins in containers 1–4 were exposed to the challenge material. For virus exposure, eight gill arches from one SGPV‐positive fish of about 160 g were cut into halves and divided equally between the four containers. The challenge material was removed after 24 h, and the alevins in containers 1–3 were re‐exposed to the stressors as described earlier to guarantee fish stress before and after virus exposure. Containers 5 and 6 served as negative controls, containing virus‐free gill material and no challenge material respectively. Five alevins from each group were sampled 3 h after the first stress exposure, after 27 h and 8 days post first stress exposure for qPCR and RT‐qPCR.

### 2.6 Experiment 3A: Alevins Exposed to SGPV‐Infected Material Stored at Various Freezing Temperatures

We also compared the SGPV infectivity of CM stored at different temperatures and for different time periods. For this purpose, a total number of 300 alevins (560 degree‐days) were put into six 1‐L Erlenmeyer flasks (50 fish in each) kept at 6°C. Alevins in flasks 1 and 2 were exposed to gills dissected from CM1 (*n* = 8) and CM2 (*n* = 3) stored at − 20°C for more than 1 year and had median SGPV Ct values of 23.7 (range 21.8–24.5) and 23.0 (range 22.1–24.4) respectively. CM3 was shipped from an SGPVD outbreak to the research facility overnight on ice, and from 12 of these fish, gills with a median SGPV Ct value of 22.0 (range 20.9–24.4) were dissected and divided into three equal portions. While the first portion was kept unfrozen and transferred directly to flask 3, the second and third portions were kept at − 80°C overnight. The following day, the second portion was transferred to the alevins in flask 4, and the third portion was gently thawed by transferring it to − 40°C for 6 h, then to − 20°C overnight and finally to 4°C for 3 h and then transferred to flask 5. Flask 6 served as a negative control. After 24 h, the challenge materials were removed from all the flasks, and the water was changed. Seven alevins were sampled at 7 and 13 days post‐exposure for qPCR, histopathology and in situ hybridisation work.

### 2.7 Experiment 3B: Cohabitation of naïve and SGPV‐Infected Alevins

The SGPV‐infected and the non‐infected alevins from Experiment 3a were used to investigate if SGPV could be experimentally transmitted from shedders to naive alevins by cohabitation. For this purpose, eight alevins exposed to the gently thawed CM3 materials in flask 5 in experiment 3a were cohabitated with 12 alevins in each of container 1 and container 2 in experiment 3a (previously exposed to CM1 and CM2, respectively, and shown to be negative for SGPV at 13 DPE). The alevins were cohabitated for 12 days and then analysed for SGPV using qPCR. To verify that SGPV was transferred from the infected alevins and not from the previous exposure of CM1 and CM2 in experiment 3b, multi‐locus variable‐number tandem‐repeat (VNTR) analysis (MLVA) was performed as described previously (Gulla et al. 2020) on all samples. Briefly, for this, DNA extracted from each sample was used as a template in a multiplex PCR featuring eight primer pairs (including one fluorescently labelled primer per pair), with subsequent capillary electrophoresis for size calling of PCR products and in silico calculation of eight‐loci MLVA profiles. A minimum spanning tree based on MLVA results was generated in BioNumerics v7.6 (Applied Maths NV, Sint‐Martens‐ Latem, Belgium).

### 2.8 Experiment 4: Alevins Exposed to Homogenised SGPV‐Infected Material Stored at − 80°C

We assessed whether gills from salmon infected with SGPV are still infective after freezing, homogenisation and centrifugation. Also, we investigated the effect of continuous water renewal (10 L/h in 12 L buckets) on the infection course and virus load.

For this purpose, gills were excised from 13 Atlantic salmon from CM3 (average SGPV Ct of 22.2; range 20.8–23.8) that had been kept at − 80°C for 6 months. While still half frozen, all four gill arches on the left side of all fish were removed and added to an Erlenmeyer flask (1 L) containing alevins (*n* = 75, 600 degree‐days; flask 1). All four gill arches on the right side were transferred to a 15‐mL ice‐cold L15 medium and homogenised with Stomacher for 2 min. Then, the homogenate was clarified by centrifugation at 4°C for 5 min at 1200 rpm, and 10 mL clarified homogenate was added to a second Erlenmeyer flask containing alevins (*n* = 75, 600 degree‐days; flask 2). The alevins were kept in the two flasks at 7°C for 24 h and transferred to two 12‐L buckets with a flow‐through system; that is, alevins from flasks 1 and 2 to buckets 1 and 2, respectively. Alevins in buckets 3 and 4 served as negative control and were exposed to whole and homogenised virus‐free gill material, respectively, for 24 h. Six alevins were sampled just after the removal of challenge material, after 24 h, 8 days and 15 days post‐exposure for qPCR and histopathology work.

### 2.9 DNA Extraction and qPCR

DNA was extracted from the alevins heads using a MagNA Pure 96 and a MagNA Pure 24 (Roche, Switzerland) automated high‐throughput instrument, following the manufacturer's Pathogen Universal Protocol. For extraction, the heads from the alevins were put into 500 μL MagNA Pure LC RNA Isolation Tissue lysis buffer (Roche, Switzerland). The heads were homogenised using 5 mm steel beads with TissueLyser II (Qiagen, Germany) at 23 Hz for 2 × 5 min. After DNA extraction, the Multiskan SkyHigh Microplate spectrophotometer (Thermo Fisher Scientific, USA) was used to estimate the purity of the DNA by calculating measurements of the absorbance ratio A260 and A280, making sure the ratio was ≥ 1.8. DNA concentrations were calculated by multiplying the absorbance at 260 nm by a factor of 50, and finally, all samples were normalised to a concentration of 50 ng/μl.

SGPV‐qPCR targeting the gene *D13L* (Gjessing et al. 2015) was performed as described by Thoen et al. (2020)) using the CFX384 Touch Real‐Time PCR Detection System (Bio‐Rad Laboratories, USA). Each DNA sample was analysed in duplicate using a standard DNA input of 100 ng (2 μL of 50 ng/μl) in a total reaction volume of 10 μL per well, with final primer concentrations of 0.4 μM, probe at 0.1 μM, MgCl_2_ at 1.5 mM, 1.6 μL nuclease‐free water and 5 μL UDG platinum supermix (Thermo Fisher Scientific, USA). The following thermocycling conditions were used: 50°C for 2 min, 95°C for 15 min, followed by 45 cycles of 94°C for 15 s, 55°C for 30 s and 72°C for 15 s. Cycle threshold (Ct) values ≥ 40 were considered negative.

### 2.10 RNA Extraction and Gene Expression Analysis

Total RNA from alevin heads was extracted on a MagNA Pure 96 instrument (Roche, Switzerland) with the MagNA Pure 96 Cellular RNA Large Volume Kit (Roche, Switzerland), using the RNA tissue FF standard cellular RNA protocol with an elution volume of 50 μL per sample. RNA yield and purity were determined by a Multiskan SkyHigh spectrophotometer (Thermo Fisher Scientific, USA). The purity of the RNA was estimated by calculating measurements of the absorbance ratio A260 and A280, making sure the ratio was ≥ 2.0. DNA concentrations were calculated by multiplying the absorbance at 260 nm by a factor of 40, and finally, all samples were normalised to a concentration of 100 ng/μl. cDNA was synthesised using the QuantiTect Reverse Transcription Kit (Qiagen, Germany) utilising 1 μg total RNA with gDNA elimination according to the manufacturer's recommendation, resulting in 20 μL cDNA.

We used RT‐qPCR to study gene expression related to stress, including the genes *CYP3A* (Arukwe 2005), *GR* (Chalmers et al. 2018), *HSP70* (Olsvik et al. 2014), *P450scc* (Arukwe 2005) and *StAR* (Arukwe 2005). *Elongation factor 1α (EF1α)* (Amundsen et al. 2021) was used as a housekeeping gene. Primer information is given in Table SS1. RT‐qPCR was performed using CFX384 Touch Real‐Time PCR Detection System (Bio‐Rad Laboratories, USA). Each sample was analysed in duplicate using a standard cDNA input of 5 ng (2 μL of 2.5 ng/μl) in a total reaction volume of 10 μL per well with primers at 10 μM, 2 μL water and 5 μL 2xSsoAdvanced Universal SYBR Green Supermix (Bio‐Rad Laboratories, USA). The following thermocycling conditions were used: initial denaturation (30 s at 95°C) followed by 40 cycles of denaturation (15 s at 95°C) and annealing/extension (30 s at 60°C). Melting curves were made by measuring the fluorescence during a temperature range of 55°C–95°C to confirm the specificity of the final amplicons, and no reverse transcriptase controls (NRT) and no‐template control (H_2_O) were used as negative controls. RT‐qPCR data were analysed using the CFX Manager Software version 3.1.1517.0823 (Bio‐Rad Laboratories, USA). The expression cycle threshold level was normalised to the *EF1α* reference gene (ΔCt), and the ΔΔCt method was used to calculate the relative expression levels and fold induction compared to samples from the control group.

### 2.11 Histopathology and In Situ Hybridisation

The entire formalin‐fixed alevins were embedded, and the orientation allowed sagittal sectioning to include a wide range of anatomical structures. Serial sections were prepared for haematoxylin and eosin (H&E) staining and in situ hybridisation using the RNAscope 2.5 HD Singleplex Red Chromogenic Reagent Kit (Advanced Cell Diagnostics Inc., Newark, CA, USA). The procedure was performed as described by Thoen et al. (2020)). Briefly, the non‐stained slides were deparaffinised in xylene and rehydrated in 100% ethanol, followed by treatment with hydrogen peroxide at room temperature for 10 min. Then, the sections were boiled in a target retrieval buffer for 15 min and incubated with protease at 40°C for 15 min. Probe hybridisation was performed using a probe targeting the SGPV *B22R1* gene, representing an early marker for SGPV infection (Amundsen et al. 2021), or the highly conserved SGPV *D13L* gene (Gjessing et al. 2015) (Advanced Cell Diagnostics, Cat. No. 1000871‐C1 and 540201). The probes were hybridised for 2 h, followed by incubation of signal amplifiers (AMP1–AMP6) according to the manufacturer's protocol (40°C for 15 or 30 min). Finally, fast red chromogen was used to visualise the hybridisation signal, before counter‐staining using Mayer's haematoxylin (Chemi Teknikk, Oslo, Norway, Cat. No. 5B‐535) diluted in distilled water 1:1 for 2 min.

### 2.12 Statistics

Wilcoxon/Kruskal–Wallis test, followed by multiple comparisons, was used to test the significance of the difference in stress genes' mRNA levels between the alevin groups exposed to different stressors at each of the different time points analysed after stress induction (Experiment 2: alevins exposed to SGPV‐infected material stored at − 20°C). Statistically significant differences in viral load between groups at the different time points in Experiments 3a (alevins exposed to SGPV‐infected material stored at various freezing temperatures) were calculated using a two‐tailed non‐parametric Mann–Whitney test. The level of significance was set at *p* < 0.05. GraphPad Prism 10.1.0 was used to make the graphs presented in this work.

## 3 Results

### 3.1 Atlantic Salmon Alevins Can Be Experimentally Infected by SGPV

In Experiment 1, alevins were exposed to non‐frozen SGPV‐infected fish or dissected gills from CM1.

**FIGURE 2:**
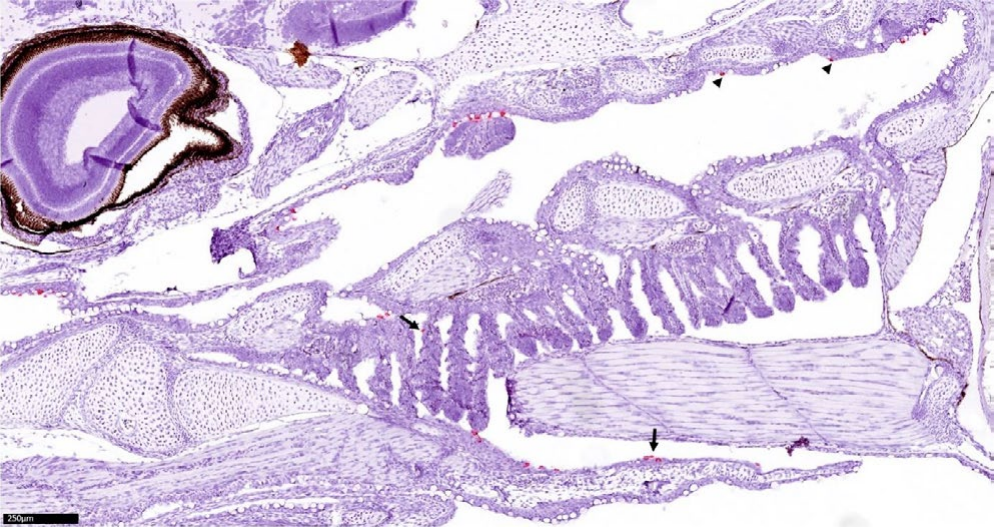
In situ hybridisation to *D13L* gene of SGPV of part of the head from Atlantic salmon alevins, exposed to gills containing SGPV and sampled 12 days post‐exposure. Red staining indicates infected cells in the gills (upper arrow), oral cavity (arrowheads) and pectoral fin (lower arrow).

At 4 and 12 days post‐exposure (DPE), few apoptotic gill epithelial cells were stained for SGPV by RNA scope in situ hybridisation (Figure 2). Some staining for SGPV was seen in epithelial cells in the gills, the oral cavity, the skin and the pectoral fins.

SGPV‐qPCR showed positive Ct values (container 1: median 29.3 and container 2: median 30.7) at 4 DPE. At 12 DPE, no significant change in virus load was recorded in either of the two containers (container 1: median 29.4 and container 2: median 29.2). Furthermore, no significant change in virus load was recorded at 25 DPE (container 1: median 28.0 and container 2: median 27.2) (Table 1). Altogether, five alevins from container 2 died, and no mortality was observed in container 1. Two of the deceased alevins were analysed for SGPV using qPCR, showing Ct values of 28.4 and 25.1. The remaining deceased alevins were preserved in formalin, but could not be evaluated due to autolysis (Table 1).

**TABLE 1:** The Atlantic salmon alevins were confirmed positive for SGPV using qPCR. SGPV Ct values (median with range) of salmon alevins exposed to SGPV using whole gill (container 1) and whole fish (container 2) after 4, 12 and 25 days post‐exposure (DPE).

DPE	Ct value median (range) container 1—whole gills	Ct value median (range) container 2—whole fish
**4**	29.3 (28.5–29.7)	30.8 (28.5–31.9)
**12**	29.4 (27.4–30.0)	29.2 (26.5–30.4)
**25**	28.0 (27.6–30.3)	27.2 (26.1–28.3)

### 3.2 No SGPV Infection Was Confirmed in Stressed Alevins Exposed to Challenge Material Stored at − 20°C

In Experiment 2, the CM1 was used as challenge material as it was confirmed to be infective in Experiment 1. CM1 had now been stored at − 20**°**C for 3 months.

**FIGURE 3:**
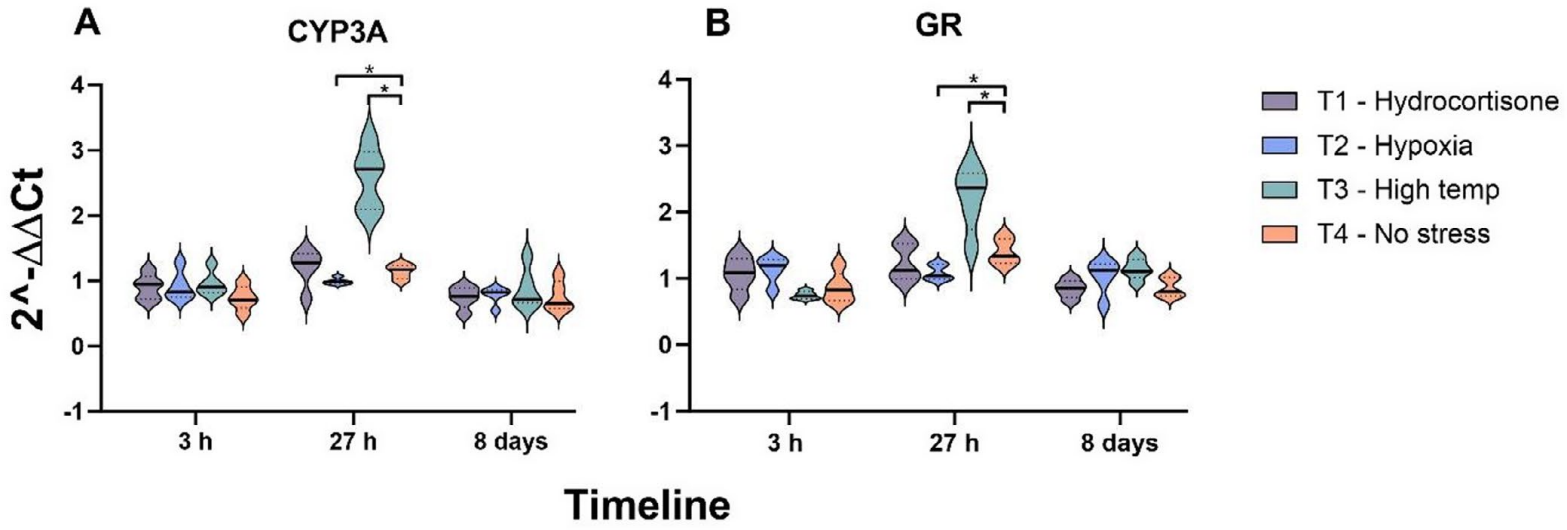
Gene expression of *CYP3A* and *GR* from alevins exposed to high temperature and hypoxia. Violin plots display the difference in expression of Atlantic salmon *CYP3A* (A) and *GR* (B) genes in alevins (*n* = 5) between fish exposed to hydrocortisone in water, low oxygen level and high temperature at 3 and 27 h, and 8 days after exposure to first stress as compared to the non‐stressed alevins. The data points represent the relative gene expression, and the plot lines depict the median and quartile values. The *p* value was calculated with the significance level set at *p < 0.05.

Alevins from all groups at 8 DPE were confirmed to be negative for SGPV using qPCR. To confirm that alevins exposed to different stress conditions responded as expected, the mRNA levels of stress‐related genes were analysed. The results showed no significant changes in the mRNA levels of CYP3A or GR until 3 h after stress induction. However, at 27 h, alevins exposed to high temperature showed a significantly higher expression of CYP3A (*p* = 0.01) and GR (*p* = 0.03) as compared to the control group with no stress (Figure 3). In the hypoxia group, the transcript levels were significantly lower for CYP3A (*p* = 0.02) and GR (*p* = 0.03) at 27 h (Figure 3). Alevins exposed to hydrocortisone had no change in the mRNA levels of any of the tested genes. Furthermore, the expression of Hsp70, P450scc and StAR did not change in response to any of the stressors (data not shown). Altogether, two alevins from the hypoxia group and one alevin from the cortisol group died during the experiment.

### 3.3 Alevins Can Be Experimentally Infected by SGPV When Exposed to Challenge Material Stored at− 80°C

**FIGURE 4:**
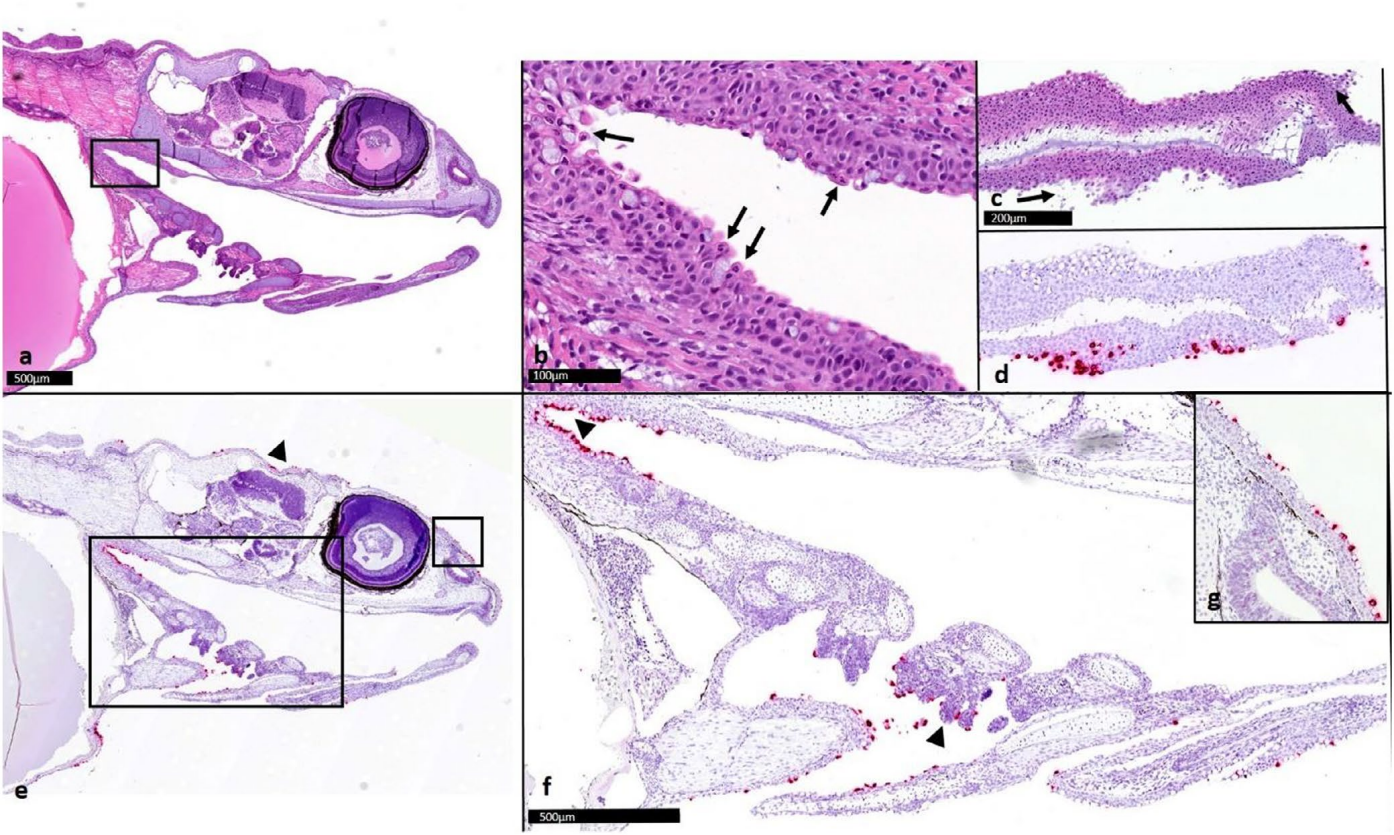
Serial section from Atlantic salmon alevins, exposed to gills containing SGPV and sampled 13 days post‐exposure. H&E staining and in situ hybridisation to *D13L* gene of SGPV. ‘b’ corresponds to the box in ‘a’. ‘f’ corresponds to the left box in ‘e’, ‘g’ corresponds to the right box in ‘e’. Arrows indicate apoptotic epithelial cells and red staining of these apoptotic cells indicates SGPV infection in tail fin (c, d), gills (f, lower arrowhead), oral cavity (e, left arrowhead) and skin on the head (e arrowhead and g).

In Experiment 3a, we wanted to pursue the negative results for SGPV in Experiment 2. We investigated if the storage temperature affected the infectivity of the challenge material. At 7 and 13 DPE, few apoptotic epithelial cells were stained for SGPV by RNA scope in the gills and oral cavity. Staining was also observed in some skin epithelial cells and was most prominent on the fins and in epithelial cells lining the dorsal part of the head (Figure 4).

**FIGURE 5:**
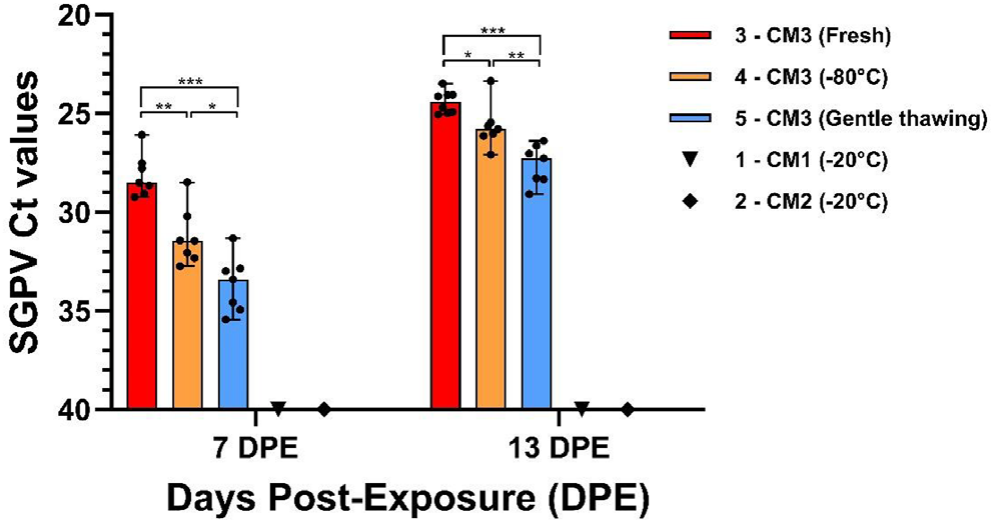
Ct values of SGPV in alevins sampled 7 and 13 days post‐exposure. SGPV Ct values (median with range) of salmon alevins (*n* = 7) exposed to SGPV by cohabitation with infected gill materials (from different outbreaks, CM1‐3) stored at different temperatures. Ct values in container 3 with fresh material at 13 days post‐exposure (DPE) show data from deceased alevins. The significant differences are calculated between containers with Icelandic challenge material (CM3). *p < 0.05, **p < 0.01, ***p < 0.001.

SGPV‐qPCR showed positive Ct values for all alevins in containers 3–5 sampled at 7 DPE (exposed to CM3; Figure 5). Alevins exposed to fresh material had significantly higher SGPV levels (*p* = 0.0070) with a median Ct of 28.5 (range 26.1–29.3), as compared to alevins exposed to challenge material stored at − 80°C (median Ct of 31.5 [range 28.5–32.7]), and to those exposed to challenge material gently thawed from − 80°C (*p* = 0.0006; median Ct of 33.4 [range 31.3–35.4]). By time, the virus replication increased in the alevins in containers 3–5, and at 13 DPE, all the alevins exposed to fresh material died. Seven of the dead individuals were picked out randomly for SGPV‐qPCR analysis and showed low Ct values with a median of 24.4 (range 23.5–25.0). The Ct values for SGPV in alevins sampled at 13 DPE in containers 4–5 had a median of 25.8 (range 23.4–27.1) and 27.3 (range 26.4–29.1) respectively (Figure 5). Samples exposed to CM1 and CM2 and samples from the control container (data not shown) tested negative for SGPV‐qPCR analysis throughout Experiment 3a (Figure 5).

### 3.4 SGPV‐Infected Alevins Can Transmit SGPV to Non‐Infected Alevins

In Experiment 3b, transmission of SGPV through cohabitation from presumed infected to presumed non‐infected alevins was tested. At 13 DPE, eight SGPV‐infected alevins (earlier exposed to gently thawed CM3 in Experiment 3a) were transferred to containers 1 and 2 with SGPV‐negative alevins (exposed previously to CM1 and CM2 in Experiment 3a with negative SGPV qPCR results).

After 11 days post‐cohabitation (DPC), seven alevins in the CM1 container and eight alevins in the CM2 container were found deceased: qPCR for SGPV of the deceased fish showed a median Ct value of 26.0 in both CM1 and CM2 containers (Table 2). The remaining alevins were euthanised the day after (12 DPC), and lower amounts of the virus as compared to the deceased alevins were detected, with median qPCR Ct values of 29.6 and 30.4 respectively (Table 2).

**TABLE 2:** SGPV infects salmon alevins via horizontal transmission. SGPV Ct values (median with range) of salmon alevin cohabitants exposed to SGPV by shedder alevins from Experiment 3a (container 5—CM 3—gentle thawing). The table shows SGPV Ct values from 11 and 12 days post cohabitation (DPC).

DPC	Ct value median (range) container 1	Ct value median (range) container 2
11	26.0 (25.5–27.4)	26.0 (24.1–26.2)
12	29.6 (27.8–30.4)	30.4 (29.7–31.8)

**FIGURE 6:**
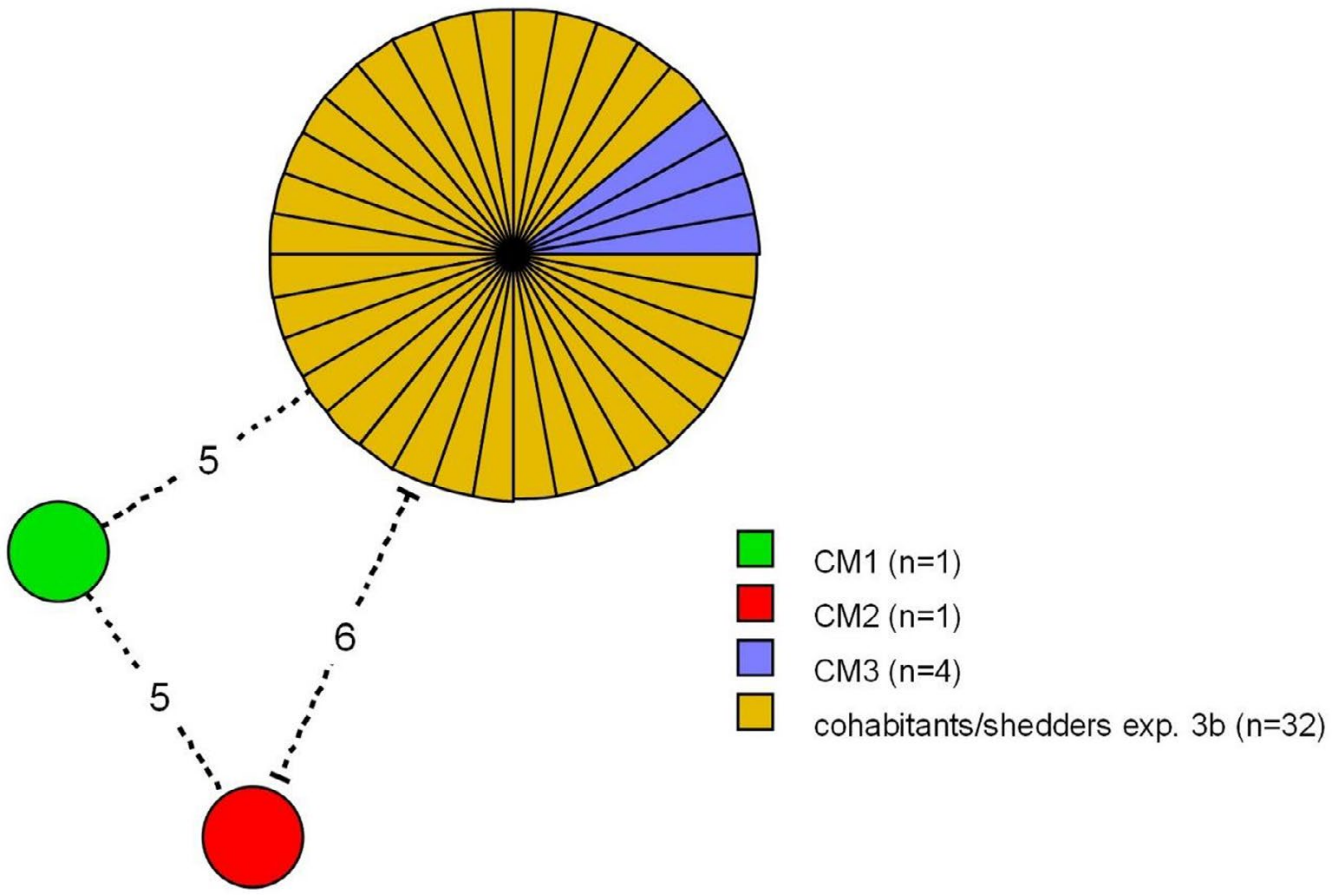
Horizontal transmission confirmed using MLVA analysis. Minimum spanning tree drawn in BioNumerics v7.6 (Applied Maths NV, Sint‐Martens‐Latem, Belgium) and visualising the genetic similarity between SGPV‐positive samples typed by a multi‐locus variable‐number tandem‐repeat (VNTR) analysis (MLVA) scheme encompassing eight VNTR loci. Samples displaying identical MLVA profiles cluster together, while numbers on connecting branches indicate how many, out of the eight VNTR loci, differ between non‐identical clusters/profiles. The Norwegian challenge materials (CM1 and CM2) are marked in green and red, while the Icelandic challenge material (CM3) is shown in blue. The alevins from the cohabitation experiment are marked in yellow.

To confirm that the SGPV infection originated from the cohabitant alevin shedders infected with CM3, rather than from prior exposure to CM1 and CM2, MLVA genotyping was conducted on the cohabitants and shredders at the end of the experiment and the challenge materials. The results showed identical MLVA profiles across all 32 analysed alevins from Experiment 3b, which also matched the CM3 profile but differed in 5–6 out of 8 VNTR loci from the CM1 and CM2 profiles (Figure 6). Eight out of 40 alevins were not analysed either because of limited DNA templates or lacking PCR products.

### 3.5 Homogenised and Whole SGPV‐Infected Gills Are Equally Infective to Alevins and Can Be Used Experimentally for Virus Challenge

In Experiment 4, we used the same material (CM3) as used in the tanks where SGPV was successfully transferred in Experiment 3. In Experiment 4, the difference in infectivity between whole SGPV‐infected gills and homogenised suspension from these was compared, and the impact of the continuous water renewal was investigated. At this time, CM3 had been kept at − 80°C for 6 months. At 8 and 15 DPE, apoptosis of a few epithelial cells was seen in the gills, oral cavity and skin, and more widespread apoptosis in the finer. Infection with SGPV was verified already at the first sampling, just after removal of the challenge material, confirmed by sparse positive staining by B22R in the gills (not shown); at that time point, the staining for D13L was negative.

SGPV‐qPCR analysis of alevins exposed to both whole infected gills and their homogenised suspension were positive in both groups. Alevins exposed to whole infected gills showed a median Ct value of 34.0 and 38.2 at 8 and 15 DPE respectively (Table 3). Interestingly, alevins exposed to gill homogenate had lower Ct values (higher virus amounts) as compared to those exposed to the whole infected gills, showing Ct values of 33.9 at both 8 and 15 DPE. No mortality was observed during the experiment. Samples from both of the control groups tested negative for SGPV throughout the experiment.

**TABLE 3:** SGPV infects salmon alevins via whole gills and gill homogenate. SGPV Ct values *(median with range)* of salmon alevins exposed to SGPV using whole gills and homogenised gills from 8 and 15 days post‐exposure (DPE).

DPE	Ct value median (range)—whole gills	Ct value median (range)—gill homogenate
**8**	34.0 (32.5–37.2)	33.9 (29.6–35.9)
**15**	38.2 (34.7–40.0)	33.9 (27.2–36.4)

## 4 Discussion

Salmon gill pox virus disease is a significant concern in the salmon farming industry (Norwegian Veterinary Institute, 2023). Since SGPV has still not been cultured, infectious material from SGPVD outbreaks is used for experimental infection challenges, and the processing and proper storage of such material are critical for the success of these studies. In this study, we studied the effect of the processing and storage of challenge material on the success of the SGPV‐controlled infection setup in salmon alevins. Logistical challenges led to the lack of replicate tanks, which is a limitation of this study, but more resources will be considered in future studies to strengthen the reliability and generalisability of the findings. Also, as for the challenge of accessing SGPV in cultivated or purified forms, which makes establishing a standard curve for virus quantitation unfeasible, we are in the process of developing a gBlock concept, which is currently under optimisation, but not yet operational. Consequently, we relied on the raw Ct values of SGPV D13L to compare viral loads between the groups in our experiments. In the context of SGPV D13L analysis, we believe that normalising or relatively quantifying the virus D13L Ct values using salmon housekeeping genes could have been misleading due to virus‐induced epithelial apoptosis—a cellular condition that can be associated with unstable expression of some housekeeping genes (Shu et al. 2019). In this study, despite the reliance on the automated DNA extraction and qPCR procedures, which we believe minimised variability in Ct values, the interpretation of raw Ct values should be confined to the specific experimental setup and conditions used.

In this study, fresh gills containing SGPV as well as gills kept at − 80°C retained infectivity when used as challenge material for salmon alevins. In contrast, material stored at − 20°C apparently lost infectivity. Previous experiments with SGPV in salmon juveniles demonstrated that when injected with hydrocortisone to simulate stress, the juveniles developed clear signs of SGPVD and exhibited high viral loads. In comparison, juveniles exposed to SGPV without hydrocortisone treatment showed no clinical signs of disease (Thoen et al. 2020). In this study, however, the modulation of stress‐related genes such as CYP3A and GR (previously associated with stress responses in salmonids) (Geslin and Auperin 2004) indicated that the absence of stress was not a contributing factor to the negative SGPV qPCR results in alevins exposed to SGPV material stored at − 20°C. This finding underscores the crucial importance of proper storage conditions for materials intended to maintain the infectivity of SGPV infection experiments.

Some poxviruses are remarkably stable in the environment, often remaining infective for decades in a dried form (Buller and Fenner 2009; Gould 1999). However, frozen virus samples may lose infectivity at higher temperatures and ice crystal formation and electrolyte imbalances can damage viral coat proteins and nucleic acids in samples stored at − 20°C (Olson et al. 2004; Pan et al. 2023; Tedeschi and De Paoli 2011). In line with this study, investigations on other viruses, including MS2 bacteriophages and herpesvirus, have demonstrated that − 80°C is ideal for preserving infectivity, with rapid cooling and thawing processes minimising viral damage compared to slower methods (Barnhart and Ash 1975; Olson et al. 2004). We demonstrated that SGPV‐infected gills used as challenge material exhibited similar infectivity in alevins as whole SGPV‐infected fish. The use of gills as a source of challenge material clearly increases the reproducibility of such experiments. Moreover, gill homogenate collected from SGPV‐infected fish was equally infective as whole infected gills and can therefore be recommended in future SGPV experimental challenges. Although the one‐to‐one comparison between the different experiments may be inappropriate, the SGPV D13L analyses revealed a general trend of low virus load (as judged by the raw Ct values) in alevins exposed to the virus in the system with a water renewal compared to those in the closed tanks. The continuous water change could have likely reduced the concentration of SGPV infectious particles in the tank water throughout the challenge period. The tendency of higher SGPV load (as judged by the lower raw Ct values) in alevins exposed to gill homogenate compared to the group exposed to whole gills could be caused by more virus particles being released from the processed gill epithelial cells from the homogenised gill tissues into the water column. Apoptosis of respiratory epithelial cells is a hallmark of SGPVD. These shedding cells contain a lot of mature virus particles (Gjessing et al., 2015; Gjessing et al. 2017), and it is likely that processing of infected gills lyses cells containing mature SGPV particles. Furthermore, challenge material in liquid form dissolves more quickly than non‐homogenised tissue. The successful use of gill homogenate is a significant finding for future infection trials, as our results suggest that its infectivity is higher compared to non‐homogenised tissue. Additionally, suspending SGPV in liquid form enables reproducibility in infection experiments, as the infectious material remains consistent across different trials.

The staining for SGPV in gill epithelial cells is in accordance with previous studies in Atlantic salmon (Thoen et al. 2020). When analysing histological sections of small fish‐like alevins, we benefit from several anatomical structures being included in the same sections. Staining for SGPV was not only seen in the gills, but also in epithelial cells covering the oral cavity as reported in a previous study (Gjessing et al., 2018) and interestingly in skin epithelial cells. In adult salmon, gas exchange mainly occurs in the gills. In alevins, however, the skin has been suggested to play a crucial role as a gas‐exchanging organ (Wells and Pinder 1996b; Zimmer et al. 2020). In newly hatched salmon, up to 80% of the O_2_ uptake is suggested to take place across the thin layer of skin and through the yolk sac (Wells and Pinder 1996a, 1996b). If the alevins' skin epithelium serves as a temporary respiratory organ until the gills are fully developed, the detection of the virus in both alevin skin and adult gills suggests that the viral infection may be mediated by one or more molecules involved in the respiratory process.

SGPV compromises the epithelial barrier in the gills both physically and immunologically (Gjessing et al., 2020) and it has been suggested that SGPV may open up for complex gill disease (Gjessing et al. 2021). It can be speculated whether the SGPV infection in the skin can facilitate secondary bacterial or fungal skin infections, as the physical weakening of the epithelial skin barrier increases vulnerability. Furthermore, the transcriptome analysis of SGPV‐infected gills suggested an immunocompromised state (Gjessing et al. 2020) and a similar process may be the case for the skin infected with SGPV and is reported for other pox viruses (Chu et al. 2011).

Performing experiments with alevins demands few resources, and no approval from the Norwegian Food Safety Authority is needed. The success of experimental SGPV infection in alevins can serve several research purposes on SGPV. The small‐sized SGPV‐infected alevins can, for example, offer a continuous virus‐shedding source for the in vitro studies aiming to establish a culturing system of the virus. The results highlight the importance of including poxvirus testing in the salmon hatcheries. Alevins with a potential sub‐clinical poxvirus infection can be suggested as a possible origin of virus outbreaks in the salmon‐rearing facilities at later stages of the salmon life cycle. In summary, this study demonstrates that Atlantic salmon alevins can be infected by SGPV, with viral presence confirmed in the gill, oral cavity and skin epithelial cells. Our findings indicate that maintaining the virus at − 80°C is crucial for preserving its infectivity, underscoring the importance of storage conditions in future SGPV research and control measures. These insights could enhance our understanding of SGPV transmission in hatchery settings and inform disease management practices.

## Author Contributions

**Marit Måsøy Amundsen:** data curation, formal analysis, writing – original draft, methodology, writing – review and editing, investigation, validation. **Haitham Tartor:** formal analysis, investigation, writing – review and editing, methodology. **Kristrun Kristþórsdóttir:** writing – review and editing. **Snorre Gulla:** formal analysis, writing – review and editing, investigation. **Brit Tørud:** investigation, writing – review and editing. **Simon Weli:** writing – review and editing. **Mona Cecilie Gjessing:** conceptualization, data curation, formal analysis, visualization, writing – original draft, methodology, investigation, supervision, project administration, writing – review and editing, funding acquisition, validation.

## Conflicts of Interest

The authors declare no conflicts of interest.

## Supporting information


**Figure S1.** A detailed follow‐up of the oxygen concentration and temperature in Experiment 2. The graphs illustrate the oxygen concentration (mg/L; panels A and C) and temperature (°C; panels B and D) within the Experiment 2 fish tanks during the 5 h of the first (A and B) and second (C and D) stress induction procedures.

**Table S1.** Probe and primer sequences used for qPCR and RT‐qPCR assays.

## Data Availability

The data that supports the findings of this study are available in the Supporting Information of this article.
