# Supplementary figures and images for: Atlantic Salmon Alevins Experimentally Exposed to Salmon Gill Poxvirus Become Infected, With the Virus Targeting Epithelial Cells in the Gills, Oral Cavity and Skin
Source: J Fish Dis. 2025 Apr 5;48(10):e14127. doi: 10.1111/jfd.14127 (PMC12421792; doi:10.1111/jfd.14127)

First stress exposure

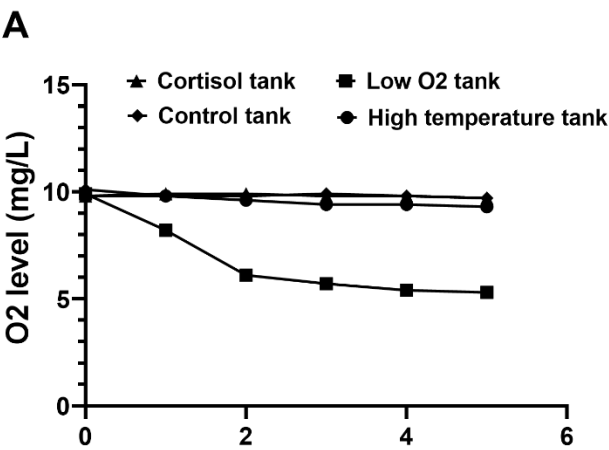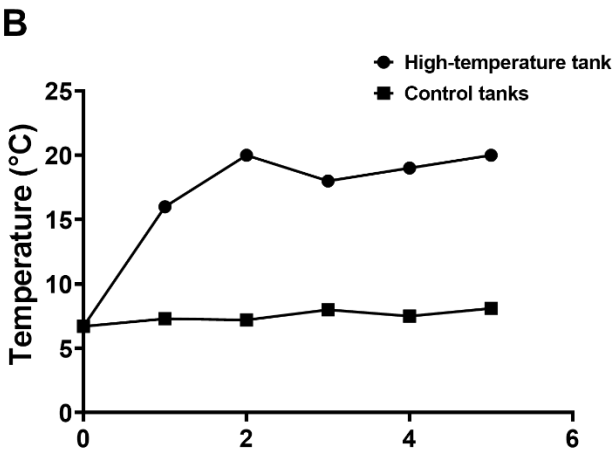

Second stress exposure

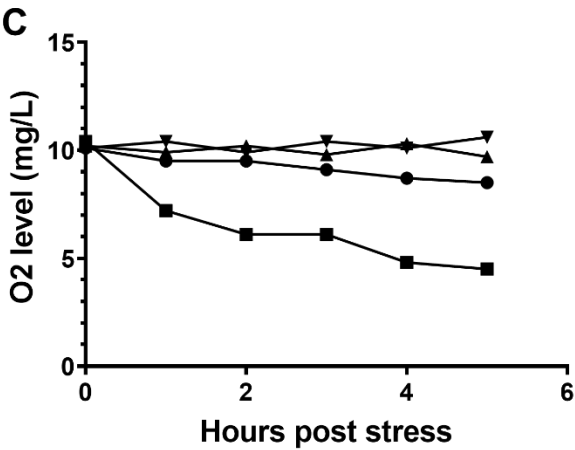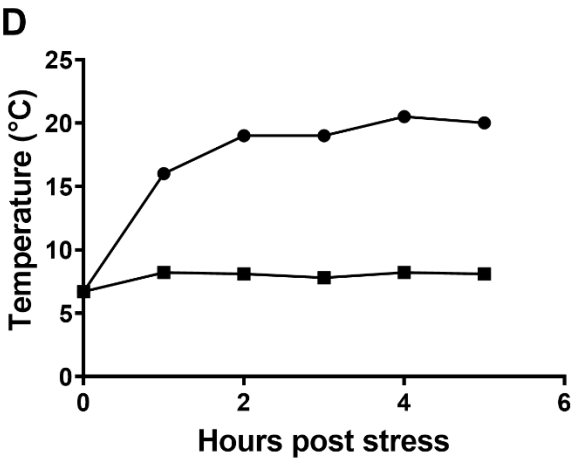

Supplement: Supplementary file 1 — Figure S1. A detailed follow‐up of the oxygen concentration and temperature in Experiment 2. The graphs illustrate the oxygen concentration (mg/L; panels A and C) and temperature (°C; panels B and D) within the Experiment 2 fish tanks during the 5 h of the first (A and B) and second (C and D) stress induction procedures. [file JFD-48-e14127-s002.zip › jfd14127-sup-0003-Supplementary figure 1.pdf]
