# Supplementary material for: Atlantic Salmon Alevins Experimentally Exposed to Salmon Gill Poxvirus Become Infected, With the Virus Targeting Epithelial Cells in the Gills, Oral Cavity and Skin
Source: J Fish Dis. 2025 Apr 5;48(10):e14127. doi: 10.1111/jfd.14127 (PMC12421792; doi:10.1111/jfd.14127)
Supplement: Supplementary file 2 — Table S1. Probe and primer sequences used for qPCR and RT‐qPCR assays. [file JFD-48-e14127-s001.zip › jfd14127-sup-0002-Legend Supplementary table 1.docx]

Supplementary table 1: Probe and primer sequences used for qPCR and RT-qPCR assays
